# Supplementary figures and images for: Combination of triciribine and p38 MAPK inhibitor PD169316 enhances the differentiation effect on myeloid leukemia cells
Source: PLoS One. 2024 Dec 31;19(12):e0312406. doi: 10.1371/journal.pone.0312406 (PMC11687802; doi:10.1371/journal.pone.0312406)

## Slide 1
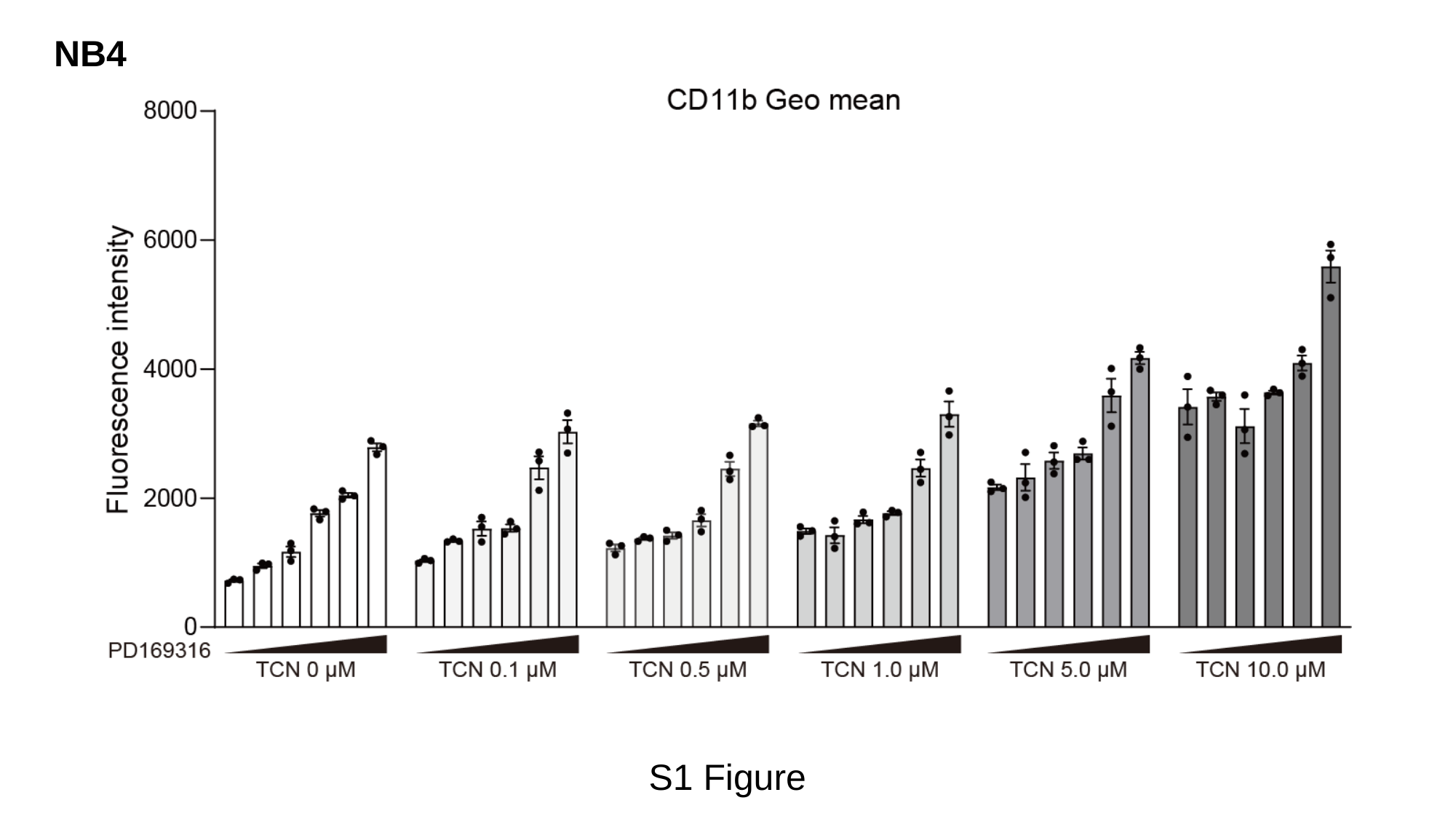

NB4
S1 Figure

Supplement: S1 Fig — The concentration of each reagent was 0, 0.1, 0.5, 1.0, 5.0 and 10 μM. The bar graphs show the expression levels of the CD11b surface markers in terms of the geometric mean fluorescence intensity. Data are presented as the mean±standard error (SE) (n = 3/group). (PPTX) [file pone.0312406.s001.pptx]

## Slide 1
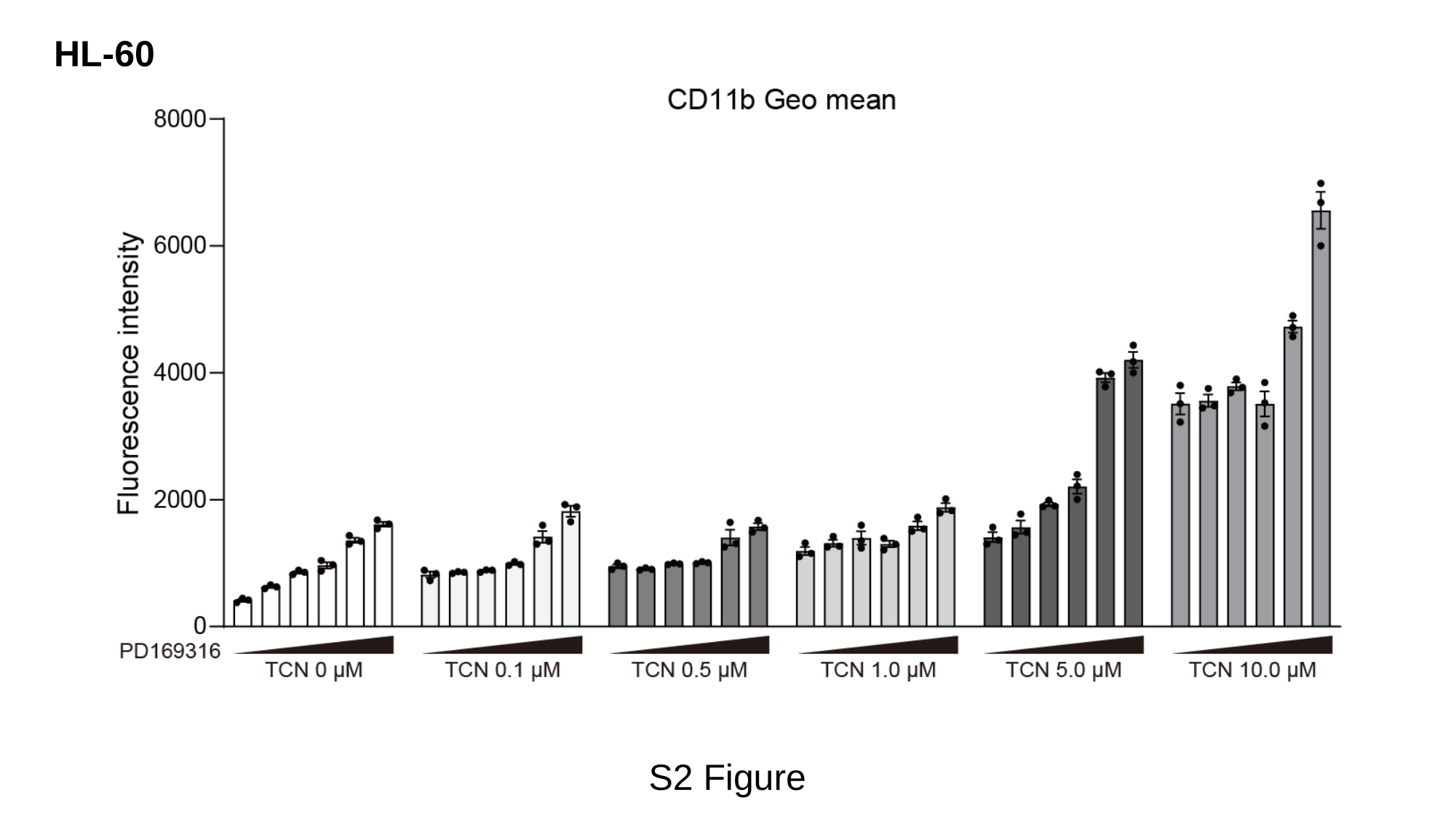

HL-60
S2 Figure

Supplement: S2 Fig — Similar to S1 Fig. (PPTX) [file pone.0312406.s002.pptx]

## Slide 1
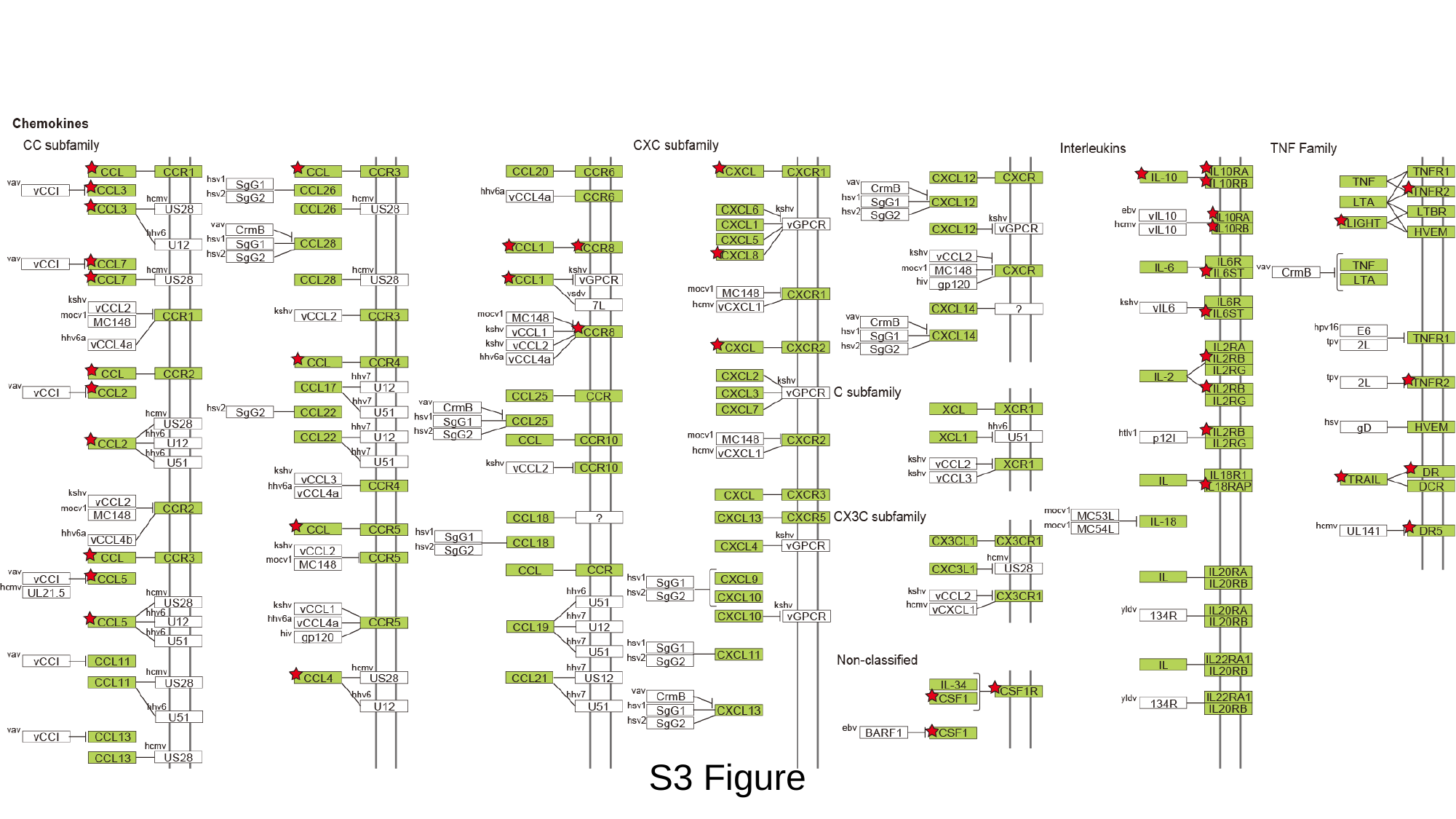

S3 Figure

Supplement: S3 Fig — The 510 genes in “viral protein interaction with cytokine and cytokine receptor” were evaluated. The red stars indicate genes with expressions induced by >5-fold by TCN and PD169316 compared with the control. (PPTX) [file pone.0312406.s003.pptx]

## Slide 1
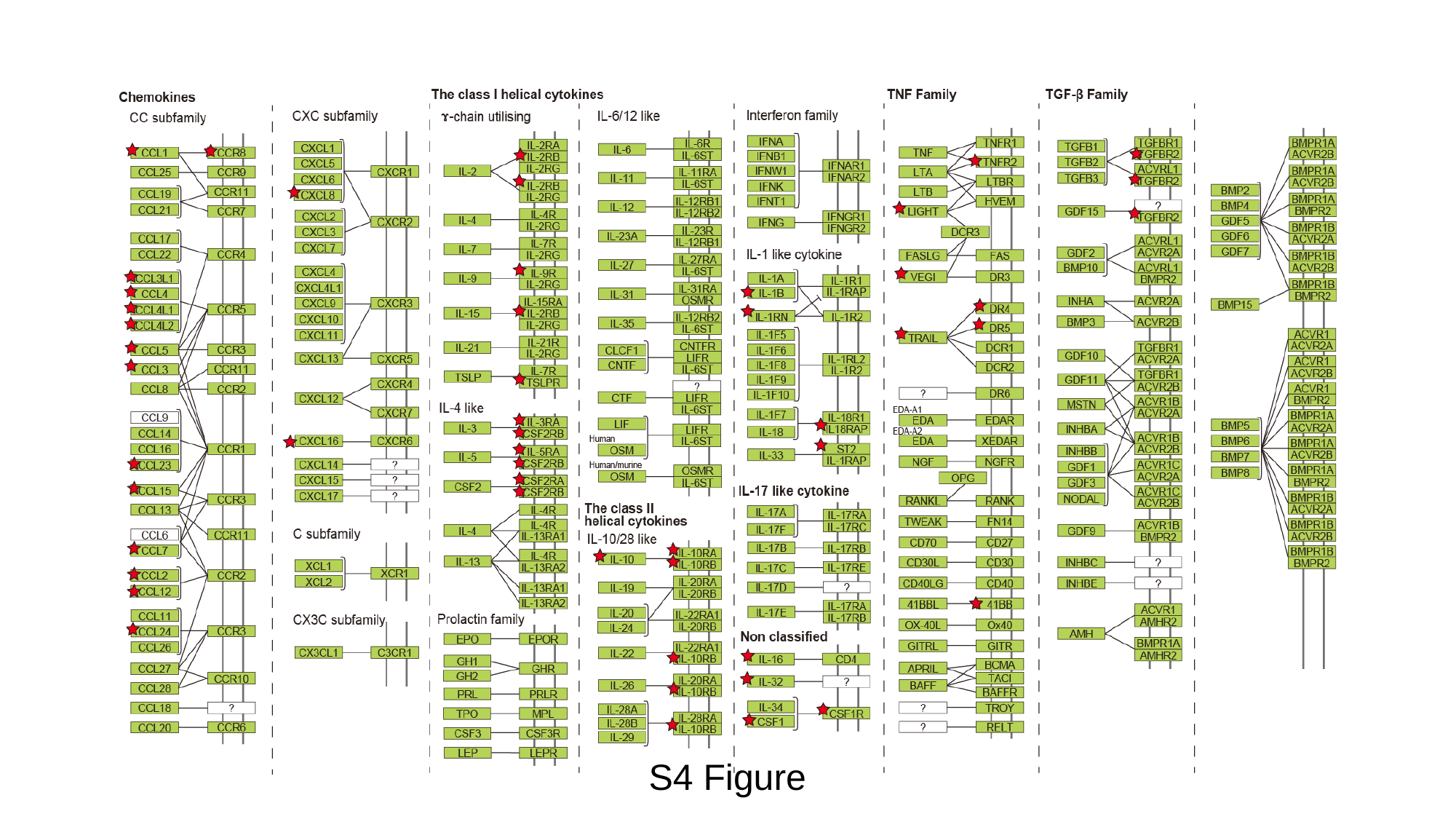

S4 Figure

Supplement: S4 Fig — The 510 genes in “cytokine-cytokine receptor interaction” were evaluated. The red stars indicate genes with expressions induced by >5-fold by TCN and PD169316 compared with the control. (PPTX) [file pone.0312406.s004.pptx]
