# Supplementary material for: Combination of triciribine and p38 MAPK inhibitor PD169316 enhances the differentiation effect on myeloid leukemia cells
Source: PLoS One. 2024 Dec 31;19(12):e0312406. doi: 10.1371/journal.pone.0312406 (PMC11687802; doi:10.1371/journal.pone.0312406)
Supplement: S1 Raw images — (PDF) [file pone.0312406.s009.pdf]

NB4

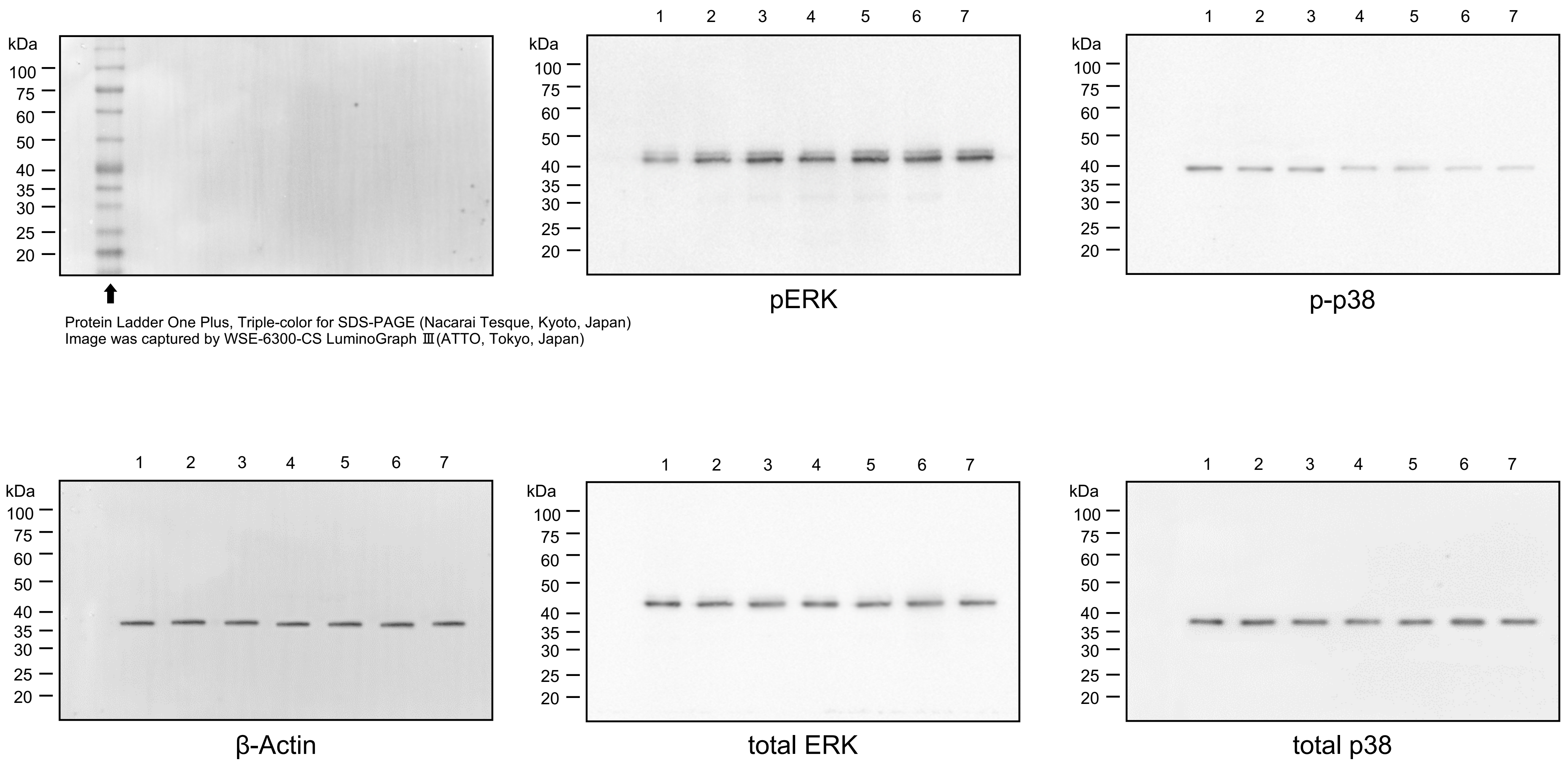

1: Ctrl 2: TCN 8h 3: PD169316 8h 4: TCN+PD169316 8h 5: TCN 24h 6: PD169316 24h 7: TCN+PD169316 24h

HL-60

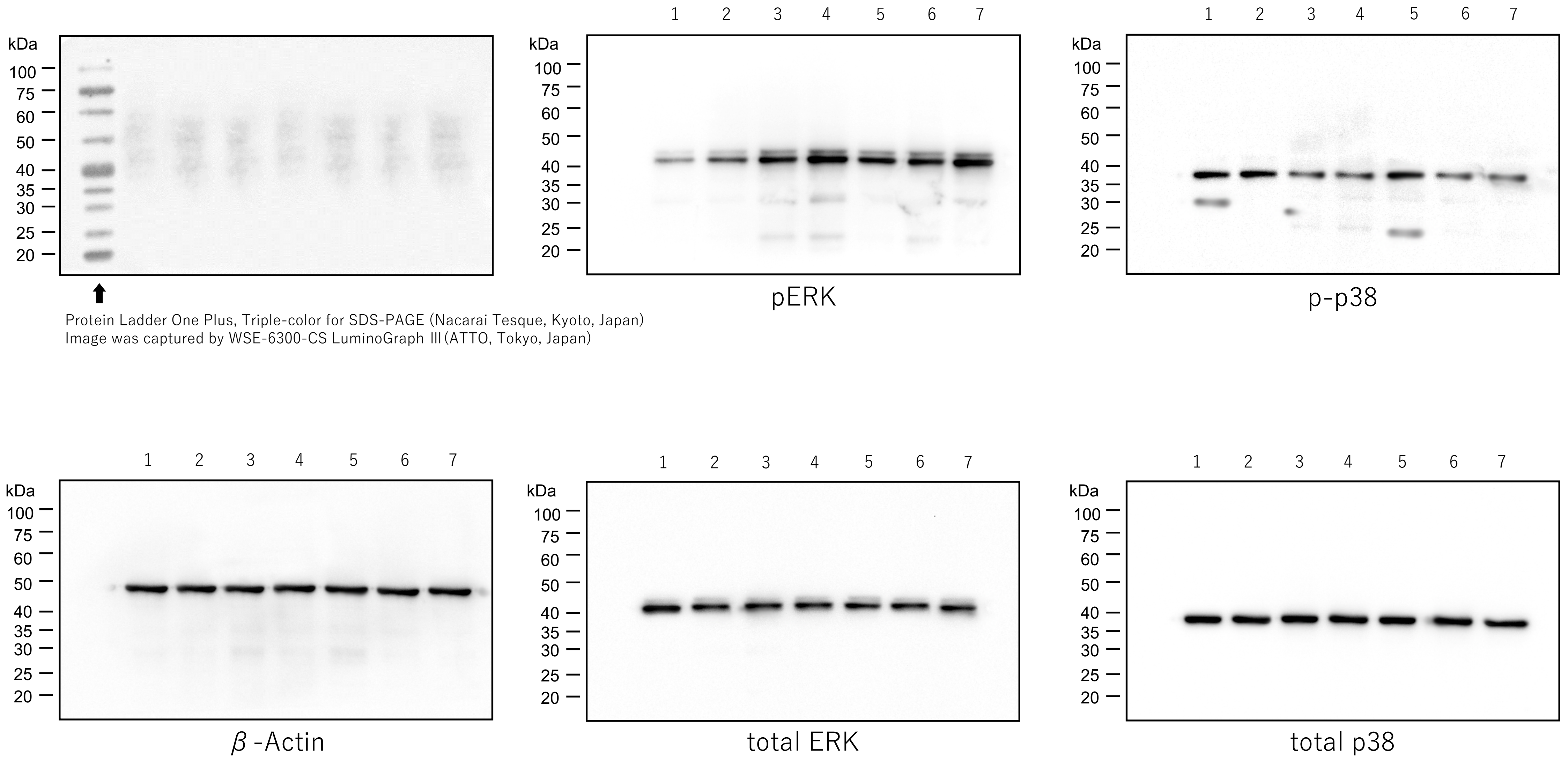

1: Ctrl　　2: TCN 8h　　3: PD169316 8h　　4: TCN+PD169316 8h　　5: TCN 24h　　6: PD169316 24h　　7: TCN+PD169316 24h
